# The effect of intensive praziquantel administration on vaccine-specific responses among schoolchildren in Ugandan schistosomiasis-endemic islands (POPVAC A): an open-label, randomised controlled trial

**DOI:** 10.1016/S2214-109X(24)00280-8

**Published:** 2024-10-16

**Authors:** Gyaviira Nkurunungi, Jacent Nassuuna, Agnes Natukunda, Ludoviko Zirimenya, Bridgious Walusimbi, Christopher Zziwa, Caroline Ninsiima, Joyce Kabagenyi, Prossy N Kabuubi, Govert J van Dam, Paul L A M Corstjens, John Kayiwa, Moses Kizza, Alex Mutebe, Esther Nakazibwe, Florence A Akello, Moses Sewankambo, Samuel Kiwanuka, Stephen Cose, Anne Wajja, Pontiano Kaleebu, Emily L Webb, Alison M Elliott, POPVAC trial team, Mirriam Akello, Florence A Akello, Hellen Akurut, Susan Amongi, Rebecca Amongin, Barbara Apule, Stephen Cose, Emmanuella Driciru, Alison M Elliott, Joyce Kabagenyi, Joel Kabali, Grace Kabami, Prossy N Kabuubi, Ayoub Kakande, Pontiano Kaleebu, Charity Katushabe, John Kayiwa, Samuel Kiwanuka, Fred Kiwudhu, Robert Kizindo, Moses Kizza, Christine Kukundakwe, Alex Mutebe, Esther Nakazibwe, Loyce Namusobya, Milly Namutebi, Christine Nankabirwa, Beatrice Nassanga, Jacent Nassuuna, Agnes Natukunda, Doreen Nayebare, Caroline Ninsiima, Ronald Nkangi, Gyaviira Nkurunungi, Denis Nsubuga, Ruth Nyanzi, Gloria Oduru, Caroline Onen, Joel Serubanja, Moses Sewankambo, Josephine Tumusiime, Pius Tumwesige, Anne Wajja, Bridgious Walusimbi, Emily L Webb, Ludoviko Zirimenya, Christopher Zziwa

**Affiliations:** a Immunomodulation and Vaccines Focus Area, Vaccine Research Theme, Medical Research Council/Uganda Virus Research Institute and London School of Hygiene and Tropical Medicine Uganda Research Unit, Entebbe, Uganda; b Department of Infection Biology, London School of Hygiene & Tropical Medicine, London, UK; c International Statistics and Epidemiology Group, Department of Infectious Disease Epidemiology, London School of Hygiene & Tropical Medicine, London, UK; d Department of Clinical Research, London School of Hygiene & Tropical Medicine, London, UK; e Department of Parasitology, Leiden University Medical Center, Leiden, Netherlands; f Department of Cell and Chemical Biology, Leiden University Medical Center, Leiden, Netherlands; g Department of Arbovirology, Uganda Virus Research Institute, Entebbe, Uganda; h Department of Global Health and Amsterdam Institute for Global Health and Development, Amsterdam University Medical Centers, Amsterdam, Netherlands

## Abstract

**Background:**

Vaccine responses differ between populations and are often impaired in rural and low-income settings. The reasons for this are not fully understood, but observational data suggest that the immunomodulating effects of parasitic helminths might contribute. We hypothesised that *Schistosoma mansoni* infection suppresses responses to unrelated vaccines, and that suppression could be reversed—at least in part—by intensive praziquantel administration.

**Methods:**

We conducted an open-label, randomised controlled trial of intensive versus standard intervention against *S mansoni* among schoolchildren aged 9–17 years from eight primary schools in Koome islands, Uganda. Children were randomly allocated to either an intensive group or a standard group with a computer-generated 1:1 randomisation using permuted blocks sizes 4, 6, 8, and 10. Participants in the intensive group received three praziquantel doses (approximately 40 mg/kg) 2 weeks apart before first vaccination at week 0, and every 3 months thereafter. Participants in the standard group were given one dose of approximately 40 mg/kg praziquantel after the week 8 primary endpoint. Participants in both groups received the BCG vaccine (Serum Institute of India, Pune, India) at week 0; the yellow fever (Sanofi Pasteur, Lyon, France), oral typhoid (PaxVax, London, UK), and first human papillomavirus (HPV) vaccination (Merck, Rahway, NJ, USA) at week 4; and the HPV booster and tetanus–diphtheria vaccine (Serum Institute of India) at week 28. The primary outcome was vaccine response at week 8 (except for tetanus and diphtheria, which was assessed at week 52). The primary analysis population was participants who were infected with *S mansoni* at baseline, determined retrospectively using either plasma circulating anodic antigen (CAA) or stool PCR. The safety population comprised all randomly allocated participants. The trial was registered at the ISRCTN Registry (ISRCTN60517191) and is complete.

**Findings:**

Between July 9 and Aug 14, 2019, we enrolled 478 participants, with 239 children per group. 276 (58%) participants were male and 202 (42%) participants were female. Among participants who were positive for *S mansoni* at baseline (171 [72%] in the intensive group and 164 [69%] in the standard group) intensive praziquantel administration significantly reduced pre-vaccination infection intensity (to median 30 CAA pg/mL [IQR 7–223] *vs* 1317 [243–8562], p<0·001) compared with standard treatment. Intensive praziquantel administration also reduced week 8 HPV-16-specific IgG response (geometric mean ratio 0·71 [95% CI 0·54–0·94], p=0·017), but had no effect on other primary outcomes. Among all participants (regardless of *S mansoni* status at baseline) intensive praziquantel administration significantly improved week 8 BCG-specific IFNγ ELISpot response (1·20 [1·01–1·43], p=0·038). Recognised adverse effects of praziquantel were reported more frequently in the intensive group. There were no recorded serious adverse events in either group.

**Interpretation:**

We show evidence suggesting that praziquantel administration improves the BCG-specific cellular response, but not humoral responses to other vaccines. Despite observational evidence that helminths impair vaccine response, these results show minimal immediate benefits of reducing helminth burden. The effect of longer-term helminth control should be investigated.

**Funding:**

UK Medical Research Council.

**Translation:**

For the Luganda translation of the abstract see Supplementary Materials section.

## Introduction

Effective vaccines are a key weapon against infectious diseases, but vaccine-specific immune responses vary between populations and are often impaired in low-income and rural settings.^1^, ^2^, ^3^ This is long-recognised for BCG, for which both vaccine response and efficacy against tuberculosis differ internationally^1^ and regionally.^4^ Oral vaccines, including rotavirus and polio vaccines, also show variable efficacy between populations.^3^ The yellow fever vaccine shows a different response in Switzerland and Uganda, with lower levels of neutralising antibody response and faster waning of response in Uganda.^2^ Within-country, influenza and tetanus vaccine responses differ between urban and rural Gabon.^5^, ^6^ New candidate vaccines for tuberculosis,^7^ malaria,^8^ and Ebola^9^ also show lower immune responses in Africa than in Europe or the USA. Previous exposure to the target pathogen or to related organisms might contribute to this phenomenon, but recent analyses implicate broader environmental sensitisation,^4^ the drivers of which have not been determined. Previous exposure is unlikely to explain the results for vaccines against rare organisms, such as the Ebola virus. Thus, drivers of population differences in vaccine response are not fully elucidated; improved understanding is important for effective vaccine development and implementation.

**Research in context**

**Evidence before this study**

On Aug 23, 2023, we used the Ovid interface to search literature from MEDLINE, Embase, Global Health, Scopus, and Web of Science, using the search terms “WHO-licenced vaccines” AND “helminths” AND (“vaccination” OR “immunisation”) in English from database inception (full keywords in appendix 2 p 18). A systematic review published by Natukunda and colleagues in 2022 identified 37 studies in humans and 24 studies in animals exploring the effect of helminths and their treatment on immune responses to vaccines. From Jan 31, 2022 (the end date of the systematic review) until Aug 23, 2023, an additional three studies in humans and four in animals had been published. Studies in animal models consistently show adverse effects of established helminth infections on responses to several unrelated vaccines; observational studies in human populations also show adverse associations. However, previous trials in humans to test whether treatment of helminths improves vaccine responses in the same individuals have been small, and inconclusive.

**Added value of this study**

Focusing on schistosomiasis, we conducted a large randomised controlled trial of intensive versus standard praziquantel administration among schoolchildren in an area where *Schistosoma mansoni* is particularly prevalent in Uganda. Participants received a portfolio of vaccines comprising live, inert, oral, and parenteral vaccines for BCG, yellow fever, oral typhoid (Ty21a), human papillomavirus (HPV), and tetanus and diphtheria. We found evidence of a small beneficial effect of intensive praziquantel administration on the immediate response to BCG, and an adverse effect on the HPV-16-specific response, but no effect on the antibody responses to other vaccine antigens. Secondary analysis suggested that the adverse effect on the HPV response may be restricted to male children. Furthermore, there was some evidence that intensive praziquantel administration had a beneficial effect on the response to oral typhoid vaccination in female participants. Before POPVAC A, no well-powered trials in adolescents or adults had been conducted to evaluate reversibility of effects of helminths on vaccine responses.

**Implications of all the available evidence**

Taken together, there is substantial evidence from published literature that schistosomiasis is associated with impaired responses to several vaccines, but little evidence, including that from our current Article, that treatment of current infection substantially improves vaccine responses. Further research should investigate the mechanisms by which exposure to helminth infections alter the immune system and modify vaccine responses, and how this can be addressed to improve vaccine responses in affected communities.

The Population Differences in Vaccine Responses (POPVAC) studies were designed to explore environmental exposures that might explain population differences in vaccine response, and whether interventions could be identified that would improve vaccine responses in rural communities in low-income settings.^10^ The focus of POPVAC A is on immunomodulation by helminth infections, specifically *Schistosoma mansoni.*^11^ Helminths have long been proposed as modulators of vaccine responses, but no well-powered trials have been conducted to evaluate reversibility of their effects in adolescents or adults.^12^

Helminths are highly prevalent in tropical and subtropical latitudes. Schistosomiasis affects over 230 million people globally, including about 25% of the Ugandan population,^13^ with peak prevalence and intensity among upper primary school aged children.^14^ Our 2022 review^12^ showed that in animal models, helminths generally impair priming and accelerate waning of vaccine responses, although effects vary by helminth species, vaccine type, and the timing of infection and vaccination. Most observational studies in humans also suggest suppressed or biased responses during helminth infection, especially during systemic infections, such as schistosomiasis and the filariases, including recent work on *S mansoni* and hepatitis B vaccination in Uganda.^15^ There is modest evidence from randomised trials that treating soil-transmitted helminth infections improves responses to the BCG^16^ or oral cholera vaccine.^17^ In five fishing communities on the Entebbe peninsula of Lake Victoria, Uganda, we found that schistosomiasis treatment improved the peak measles-booster response in children aged 3–5 years.^18^

POPVAC A was therefore designed to comprehensively address the hypothesis that *S mansoni* infection causes suppression of responses to unrelated vaccines, and that this effect can be reversed by intensive treatment with praziquantel. The trial population was selected to comprise children at intense risk of exposure to *S mansoni* infection in an area where the parasite is particularly prevalent, an island setting in Lake Victoria, Uganda. A portfolio of vaccines, of potential benefit to the children and comprising live, inert, oral, and parenteral vaccines, was provided to enable a comprehensive assessment and comparison of effects on vaccines with different characteristics.

## Methods

### Study design and participants

**Figure 1:**
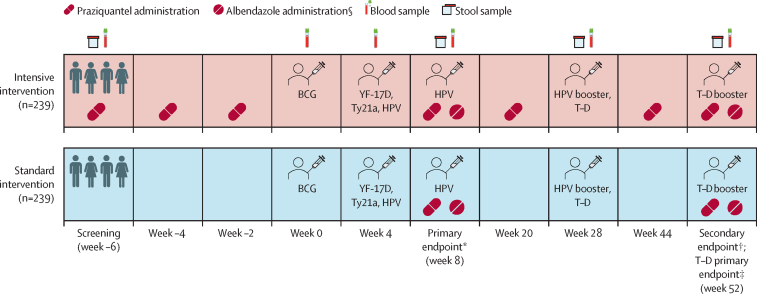
Trial schedule YF-17D=yellow fever vaccine. Ty21a=live oral typhoid vaccine. HPV=virus-like particle human papillomavirus vaccine. T–D=tetanus–diphtheria vaccine. Created with BioRender.com. *Primary endpoint following BCG, YF-17D, Ty21a, and HPV vaccination; additionally, an HPV dose was given to previously unvaccinated girls aged 14 years or older. †Secondary endpoint following BCG, YF-17D, Ty21a, and HPV vaccination. ‡A T–D boost was given to comply with the Uganda National Expanded Program on Immunization guidelines.

The trial protocol has been published previously.^11^ We conducted a two-group parallel randomised controlled trial, with individual participant randomisation to intensive or standard praziquantel intervention (figure 1). The trial was conducted in the Koome islands of Lake Victoria, Mukono district, Uganda. These *S mansoni*-endemic islands^14^ are predominantly occupied by fishing communities. The trial population comprised children aged 9–17 years who were attending primary school in years 1–6.

Participants were included if they attended one of the eight selected schools (all schools in the area barring one, which only had primary school years 1–4), were willing to provide locator information and, for female participants, if they agreed to avoid pregnancy during the trial. In our trial, sex was self-reported, with categories restricted to male and female. Exclusion criteria were history of a serious psychiatric condition; history of immunodeficiency, endocrine disorder, neurological illness, cancer, or cardiovascular, gastrointestinal, liver, or renal disease; HIV seropositivity, haemoglobin under 82 g/L, pregnancy or current lactation; history of an allergic reaction to any vaccination or allergy to any component of the trial vaccines; previous yellow fever, oral typhoid, human papillomavirus (HPV), BCG, or tetanus and diphtheria vaccination at age 5 years or older; tendency to develop keloid scars; acute illness characterised by any of the following: fever, impaired consciousness, convulsions, difficulty in breathing, or vomiting; concurrent oral or systemic steroid medication or the concurrent use of other immunosuppressive agents within 2 months before enrolment; use of an investigational medicinal product or non-registered drug, live vaccine, or medical device other than the study vaccines within 30 days before dosing with the first study vaccine, or planned use during the trial period; or administration of immunoglobulins, any blood products, or both within the 3 months preceding the planned study vaccination date.

All participants and their parents or guardians gave written informed assent and consent, respectively. Ethics approval was granted by the Uganda Virus Research Institute Research Ethics Committee (references GC/127/18/09/680 and GC/127/19/05/664), the London School of Hygiene and Tropical Medicine Observational/Interventions Research Ethics Committee (reference 16032), the Uganda National Council for Science and Technology (reference HS2486), and the Uganda National Drug Authority (reference CTA0093). The trial was registered at the ISRCTN Registry (ISRCTN60517191).

### Randomisation and masking

An independent statistician used randomly permuted blocks (sizes 4, 6, 8, and 10) to generate a randomisation code used to assign participants in a 1:1 ratio to receive either intensive or standard treatment. The randomisation code was kept confidential and only released to those responsible for providing or preparing trial interventions. To implement randomisation, opaque, sealed envelopes containing a card indicating the assigned treatment were labelled sequentially with the randomisation numbers. These numbers were successively assigned by the clinician to eligible participants at enrolment, until the desired sample size was achieved. Whenever the next randomisation number in the sequence was assigned, the envelope bearing that number was opened to reveal treatment assignment. As the trial was open label, investigators and participants were not masked to group allocation. Only immunology laboratory staff assessing trial outcomes were masked.

### Procedures

Sociodemographic and clinical details were collected from all participants at screening. Additionally, clinical details were recorded at week 0, 4, and 28 vaccination timepoints, and at the week 8 and week 52 endpoints. Figure 1 shows timepoints at which blood and stool samples were obtained.

Participants in the intensive group received three praziquantel doses (approximately 40 mg/kg, assessed by the WHO height pole^19^ which allows the estimation of doses without the requirement of weighing scales) before the first trial vaccination at week 0, each dose 2 weeks apart, with the last dose 2 weeks before the first vaccination. This was followed by one praziquantel dose at week 8, and thereafter praziquantel administration every 3 months until the end of follow-up at week 52. Participants in the standard group received their first praziquantel dose after sample collection for the primary outcome measurement at week 8, and a second dose at the last trial timepoint at week 52. In addition to the trial intervention, our published protocol allowed administration of a 400 mg single dose of albendazole in both groups (in accordance with Uganda Ministry of Health guidelines) for the 6-monthly treatment of other helminths at weeks 8, 32, and 52. However, due to Uganda's COVID-19 lockdown, the week 32 visit was omitted and therefore we amended the protocol so all participants received albendazole only at weeks 8 and 52.

Trial participants were vaccinated with the live parenteral BCG vaccine (Serum Institute of India, Pune, India; 0·1 mL intradermally, right upper arm) and yellow fever vaccine (YF-17D; Sanofi Pasteur, Lyon, France; 0·5 mL intramuscularly, left upper arm); live oral typhoid vaccine (Ty21a; PaxVax, London, UK; one capsule per day taken on 3 alternate days), quadrivalent virus-like particle HPV vaccine (Merck, Rahway, NJ, USA; 0·5 mL intramuscularly, left upper arm), and toxoid vaccines (tetanus–diphtheria; Serum Institute of India; 0·5 mL intramuscularly, left upper arm). The vaccination schedule (figure 1) consisted of three main vaccination days in weeks 0, 4, and 28. To comply with prevailing government guidelines, an additional HPV vaccination was provided at week 8 after collection of study samples for female participants aged 14 years and older who had not received HPV vaccination before this trial. For all participants, a further HPV booster was given at week 28, and a tetanus–diphtheria booster on trial completion at week 52.

The primary outcomes were BCG-specific IFNγ responses 8 weeks post-BCG vaccination; YF-17D-neutralising antibody titres at 4 weeks post-YF-17D vaccination; *Salmonella enterica* serovar Typhi (henceforth *S* Typhi) O-lipopolysaccharide (O:LPS)-specific IgG concentration at 4 weeks post-Ty21a vaccination; HPV type-16 and type-18 L1 protein-specific IgG concentration at 4 weeks post-HPV priming vaccination; and tetanus and diphtheria toxoid-specific IgG concentration at 24 weeks post-tetanus–diphtheria vaccination. Primary outcome assays were conducted at week 8 (and at week 52 for tetanus–diphtheria). Our published protocol^11^ specified assessment of baseline levels of tetanus and diphtheria toxoid-specific IgG concentration at week 28, before tetanus–diphtheria administration, and the primary outcome for tetanus–diphtheria at week 32, 4 weeks after the immunisation. However, due to Uganda's COVID-19 lockdown, the week 32 visit was omitted (including the planned praziquantel and albendazole administration), and therefore we amended the protocol so the primary outcomes for tetanus–diphtheria were assessed 24 weeks post-vaccination, at week 52 of the trial. Pre-vaccination responses for all other vaccines were assessed at week 0.

We assessed BCG-specific IFNγ responses using freshly isolated peripheral blood mononuclear cells (PBMCs) and a Human IFNγ (ALP) ELISpot Flex kit (Mabtech, Stockholm, Sweden). Assay details are documented in appendix 2 (pp 12–13). We report results as spot-forming units (SFUs) per million PBMCs, calculated sequentially by first, subtracting mean SFUs of unstimulated assay wells from mean SFUs of duplicate BCG-stimulated wells; and second, correcting for the number of PBMCs (300 000) per well. Samples that had more than 83·3 SFUs per million PBMCs in the unstimulated well were considered invalid and not included in the final analysis.

Plasma neutralising antibodies against yellow fever virus were assessed using plaque reduction neutralising reference tests (PRNTs, appendix 2 p 14). We report PRNT_50_ and PRNT_90_ titres, defined as the reciprocal of the last plasma dilution that reduced by 50% or 90%, respectively, the number of virus plaques infected by 100 plaque forming units per 0·1 mL of the reference 17D virus preparation. Plasma HPV-16-specific and HPV-18-specific IgG antibodies were measured using an ELISA, adapted from a method employed by Miller and colleagues.^20^ To measure oral typhoid vaccine-specific responses, *S* Typhi O:LPS-specific IgG levels were quantified by ELISA. Standards used in this assay were derived from a pooled sample generated from sera of known O:LPS-specific IgG titres, provided by the Oxford Vaccine Centre Biobank (Oxford, UK). These sera had been collected from the highest responders to O-antigen following challenge with *S* Typhi in a controlled human infection study.^21^ Anti-diphtheria and anti-tetanus IgG levels were also determined by ELISA, using WHO reference preparations of diphtheria toxoid (National Institute for Biological Standards and Control [NIBSC] product code 13/212) and tetanus toxoid (NIBSC product code 02/232) as assay antigens, and WHO international anti-toxin preparations for diphtheria (NIBSC 10/262) and tetanus (NIBSC 13/240) as standards. Details of the HPV-specific, *S* Typhi-specific, and tetanus and diphtheria-specific IgG assays are shown in appendix 2 (pp 15–16).

Planned secondary outcomes were vaccine response waning assessed by repeating primary outcome assays at week 52 and area under the curve (AUC) responses to all vaccines apart from tetanus–diphtheria, seropositivity and protective antibody levels following vaccination (YF-17D and tetanus toxoid), seroconversion rates (Ty21a), HPV vaccine response after priming versus after boosting dose, plasma *S mansoni* circulating anodic antigen (CAA) levels, and stool *S mansoni* PCR positivity. Participants with PRNT_50_ titres of ten or greater following YF-17D vaccination were considered seropositive.^22^ Tetanus toxoid-specific IgG levels of 0·1 international units (IUs) per mL post tetanus–diphtheria vaccination^23^ were considered protective. Seroconversion following Ty21a vaccination was defined as a ≥4-fold increase in *S* Typhi O:LPS-specific IgG over baseline.^24^ *S mansoni* infection status was determined retrospectively, after samples at all relevant timepoints had been collected through plasma measurement of CAA using the SCAA20 assay format (30 pg**/**mL positivity threshold)^25^ and stool *S mansoni* DNA detection with a multiplex PCR assay that also included primers and probes for *Necator americanus* and *Strongyloides stercoralis* DNA detection. *Plasmodium falciparum* infection status was also determined retrospectively by PCR (for assay details see appendix 2 pp 17–18). The safety population comprised all randomly allocated participants.

### Statistical analysis

Previous work suggested that standard deviations of primary outcome measures would lie between 0·3 log_10_ and 0·6 log_10_,^2^, ^26^ and that effective treatment could increase responses by approximately 0·2 log_10_.^27^ A sample size of 306 (153 per group) was determined to give over 80% power to detect absolute differences of 0·1 (for SD 0·3) to 0·2 (for SD 0·6) log_10_ units in vaccine response, corresponding to geometric mean ratios of 1·26 and 1·59, respectively. The primary analysis population was specified to be participants who were *S mansoni* positive at baseline. Based on our previous study in the same setting,^14^ we anticipated detecting infections in 80% or more of participants. Allowing for this, and 20% loss to follow-up, our total target sample size was 480 participants, 240 in each trial group, of whom 192 were anticipated to be *S mansoni* positive at baseline.

Baseline characteristics of participants, intervention fidelity and vaccine uptake were summarised by trial group. *S mansoni* prevalence and intensity at each timepoint were compared between trial groups using χ^2^ tests and Wilcoxon rank sum tests, respectively. Analysis was done by intention to treat, so that participants were included in the group to which they were randomly assigned, regardless of the number of praziquantel treatments received. All outcome comparisons were done in two analysis populations: the primary analysis population comprised participants who were *S mansoni* positive on either CAA or PCR at baseline to investigate the effect of parasite removal; the secondary analysis population was all randomised participants. For each vaccine-specific outcome, we excluded from the analysis participants who did not receive the vaccine corresponding to that outcome; for Ty21a response, we excluded participants who did not receive any of the three Ty21a vaccine doses.

Primary outcomes and continuous secondary outcomes (waning response assessed at week 52, AUC from the week 8 and week 52 responses for BCG, YF-17D, Ty21a, and HPV) were log_10_ transformed and compared between trial groups using unpaired Student's *t* tests with results back-transformed to give geometric mean ratios and 95% CIs. AUC was calculated using the trapezoidal rule and variance estimated as squared deviations from the mean. Protective immunity outcomes were summarised as proportions and compared between trial groups as differences in proportions and corresponding 95% CIs. The effect of intensive versus standard praziquantel administration on priming versus boosting for HPV was assessed among those who had received both HPV doses, using a mixed effects linear regression model for HPV response at week 8 and week 52 and including an interaction between timepoint and trial group. The subset of girls aged 14 years and older who received an additional HPV dose at week 8 were excluded from the week 52 HPV analysis. Primary analyses did not adjust for covariates, as this was a randomised trial. We did not control for corresponding pre-vaccination vaccine responses (measured at week 0), as these were potentially already impacted by trial intervention administered weeks earlier. Planned subgroup analyses assessed whether the effect of intensive versus standard intervention on primary outcomes differed by sex; this was done using linear regression and including an interaction term between trial group and participant sex. Analyses and data visualisation were done in Stata version 17.0 and GraphPad Prism version 9.0.0.

### Role of the funding source

The funder and sponsor of the trial had no role in trial design, data collection, data analysis, data interpretation, or writing of the report.

## Results

**Figure 2:**
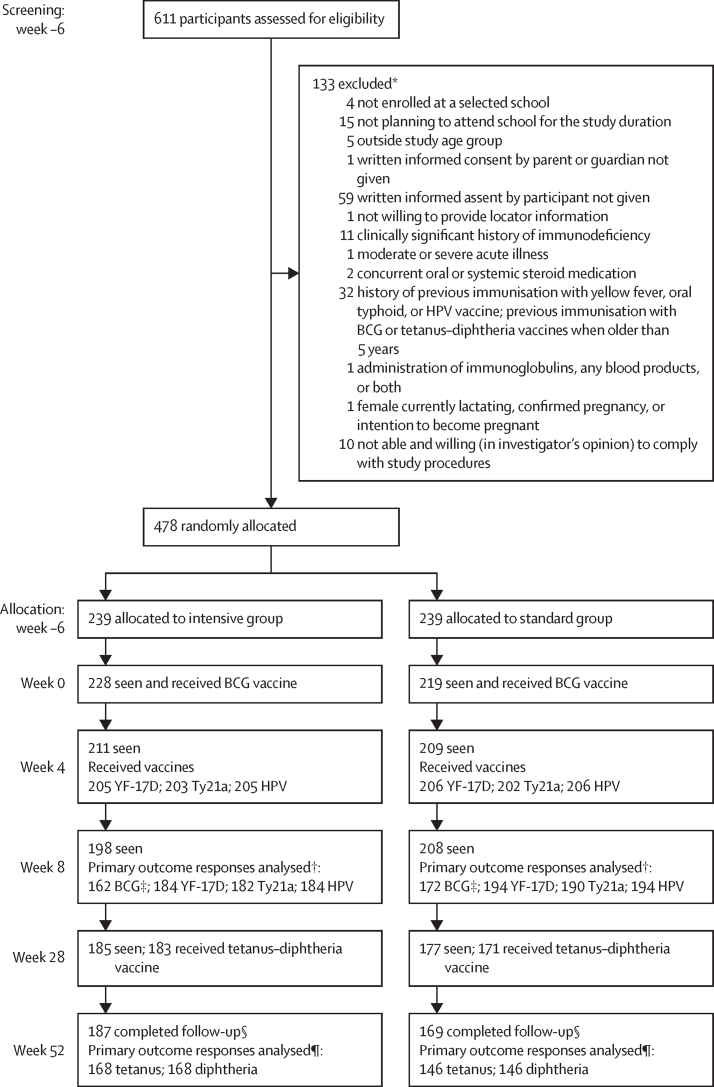
Trial profile YF-17D=yellow fever vaccine. Ty21a=live oral typhoid vaccine. HPV=virus-like particle human papillomavirus vaccine. PBMCs=peripheral blood mononuclear cells. SFU=spot-forming units. *Some participants had multiple reasons for exclusion †Reasons for not being included in the final analysis included no sample at the primary endpoint, or the sample was available, but the participant did not receive the correctly allocated vaccine. ‡BCG INFγ ELISpot assay—samples that had more than 83·3 SFUs per million PBMCs in the unstimulated well were considered invalid and not included in the final analysis. §In the intensive group, 52 participants did not complete follow-up, and 70 did not complete follow-up in the standard group, totalling 122 participants. The reasons for withdrawal were loss to follow-up (n=104), meeting exclusion criteria during follow-up (n=1) and withdrawal of consent (n=17). ¶Reasons for not being included in the final analysis were no sample at week 52, or the sample was available but participant did not receive a tetanus–diphtheria vaccine at week 28.

**Figure 3:**
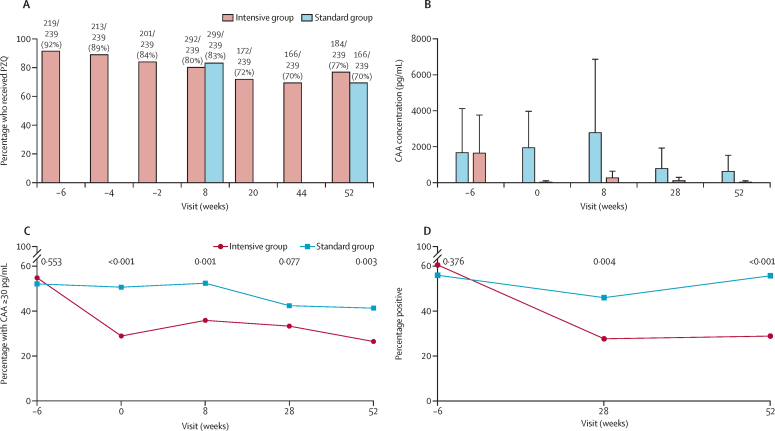
PZQ uptake and effect of treatment on *S mansoni* infection Percentage of patients who received PZQ (A), *S mansoni* intensity (B) and prevalence (C and D) at each timepoint were compared between trial groups using Wilcoxon rank sum tests and χ^2^ tests, respectively. Furthermore, linear mixed models were fitted to compare *S mansoni* intensity (B, p=0·03) and prevalence (C, p<0·0001; D, p<0·0001) between trial groups, from week –6 to week 52. CAA=circulating anodic antigen. PZQ=praziquantel. *S mansoni=Schistosoma mansoni.*

Between July 9 and Aug 14, 2019, 611 schoolchildren from eight primary schools in the Koome subcounty were assessed for eligibility, and 478 children were enrolled and randomly assigned in a 1:1 ratio to either the intensive group or standard treatment group (figure 2). 276 (58%) participants were male and 202 (42%) participants were female. 187 (78%) of 239 participants in the intensive group and 169 (71%) of 239 participants in the standard group completed follow-up at week 52. In the intensive group, primary outcome responses were analysed in 162 participants for BCG, 184 participants for YF-17D and HPV, 182 participants for Ty21a, and 168 participants for tetanus and diphtheria. For the standard group, primary outcomes were analysed in 172 participants for BCG, 194 participants for YF-17D and HPV, 190 participants for Ty21a, and 146 participants for tetanus and diphtheria). Participant baseline characteristics from screening are shown in table 1. At enrolment, 171 (72%) participants in the intensive group and 164 (69%) participants in the standard group had either *S mansoni* CAA levels of 30 pg/mL or higher, were *S mansoni* positive by PCR, or both. Praziquantel intervention uptake in the intensive group was 219 (92%) of 239 at screening (6 weeks before the first vaccination) and reduced to 201 (84%) of 239 2 weeks before the first vaccination, with 237 (99%) of the 239 participants receiving at least one praziquantel treatment before the first vaccination (figure 3A and appendix 2 p 3). Intervention uptake was not significantly different between the two groups at weeks 8 and 52 post-BCG vaccination, when both groups received praziquantel treatment (p=0·41 and 0·06, respectively). Adverse events recorded among participants in each intervention group are shown in appendix 2 (pp 3–4): recognised adverse effects of praziquantel were reported more frequently in the intensive group. There were no recorded serious adverse events in either intervention group.

**Table 1:** Baseline demographic and clinical characteristics

		**Intensive group (n=239)**	**Standard group (=239)**
Age in years, median (IQR)		12 (10–13)	11 (10–13)
Sex			
	Male	140 (59%)	136 (57%)
	Female	99 (41%)	103 (43%)
School			
	Nanyonyi Nursery and Primary School	12 (5%)	13 (5%)
	Bethel Junior Day and Boarding School	22 (9%)	24 (10%)
	Buyana Roman Catholic Primary School	79 (33%)	77 (32%)
	Ddamba Parents Primary School	34 (14%)	35 (15%)
	Koome Church of Uganda Primary School	36 (15%)	34 (14%)
	Happy Saints Nursery and Primary School	7 (3%)	9 (4%)
	Kimmi Nursery and Primary School	19 (8%)	19 (8%)
	Good Hope Day and Boarding Nursery and Primary School	30 (13%)	28 (12%)
Helminth infections			
	*S mansoni*, CAA ≥30 pg/mL	131/239 (55%)	124/238 (52%)
	*S mansoni*, PCR positive	144/238 (61%)	133/237 (56%)
	*S mansoni*, CAA ≥30 pg/mL or PCR positive	171/239 (72%)	164/239 (69%)
	*N americanus*, PCR positive	54/238 (23%)	57/237 (24%)
	*S stercoralis*, PCR positive	18/238 (8%)	20/237 (8%)
Malaria infection, PCR positive for *P falciparum*		39/239 (16%)	48/239 (20%)

CAA=circulating anodic antigen. *N americanus*=*Necator americanus*. *P falciparum*=*Plasmodium falciparum*. *S mansoni*=*Schistosoma mansoni*. *S stercoralis*=*Strongyloides stercoralis*.

228 (95%) participants in the intensive group and 219 (92%) participants in the standard treatment group received the first vaccination with BCG at week 0. At week 4, vaccine uptake in the intensive versus the standard group was as follows: YF-17D 86% versus 86%; first dose of Ty21a 84% versus 85%; and HPV 86% versus 86%. At week 28, 77% and 72% of enrolled participants in the intensive and standard treatment groups, respectively, received a tetanus–diphtheria vaccination. Full details on vaccine uptake are shown in appendix 2 (p 4).

Baseline concentrations of *S mansoni* CAA were balanced between intervention groups (table 1, figure 3B). Among participants who were positive for *S mansoni* at baseline (CAA ≥30 pg/mL), the intensive treatment, compared with standard intervention, significantly reduced *S mansoni* infection intensity (median CAA 30 pg/mL, [IQR 7–223] *vs* 1317 [243–8562], p<0·0001; figure 3B) and prevalence by week 0 (p<0·001), but did not eliminate *S mansoni* infection in a substantial number of participants (figures 3C, D). Pre-vaccination vaccine-specific responses were similar between intervention groups (appendix 2 p 4).

The effect of intensive versus standard praziquantel administration on vaccine-specific responses is shown in table 2 and appendix 2 pp 8–9. In participants who had *S mansoni* CAA of 30 pg/mL or higher or who were PCR positive at baseline (primary analysis population), intensive praziquantel administration had no statistically significant effect on BCG-specific IFNγ ELISpot response, yellow fever PRNT antibody titres, *S* Typhi O:LPS-specific, HPV-18-specific, or tetanus and diphtheria toxoid-specific antibody response at the primary endpoint for each vaccine compared with standard treatment, but had a significant adverse effect on the HPV-16-specific IgG response, with a geometric mean ratio (GMR) of 0·71 (95% CI 0·54–0·94), p=0·017. Considering all participants who were randomly allocated, the secondary analysis population, intensive praziquantel administration improved the week 8 BCG-specific IFNγ ELISpot response (GMR 1·20 [1·01–1·43], p=0·038) compared with standard treatment. There was no effect on responses to the other vaccines. At the secondary endpoint (week 52 post-BCG vaccination), there was no effect of intensive, compared with standard, praziquantel administration on vaccine responses in either the primary or the secondary analysis populations.

**Table 2:** Effect of intensive versus standard praziquantel administration on vaccine responses

		**Primary analysis: CAA ≥30 pg/mL or PCR positive at baseline**				**Secondary analysis: all participants**			
		n*	Geometric mean (SE)	Geometric mean ratio (95% CI)	p value	n*	Geometric mean (SE)	Geometric mean ratio (95% CI)	p value
**Primary endpoint analysis**									
BCG-specific IFNγ (8 weeks post-vaccination), SFUs per 1 million PBMCs									
	Intensive group	110	209·37 (1·08)	1·17 (0·95–1·43)	0·14	162	218·72 (1·06)†	1·20 (1·01–1·43)†	0·038†
	Standard group	110	179·42 (1·08)	ref	..	172	181·77 (1·07)†	ref	..
Yellow fever PRNT_50_ titres (4 weeks post-vaccination)									
	Intensive group	133	1445·27 (1·11)	0·89 (0·66–1·20)	0·44	184	1441·24 (1·10)	0·97 (0·74–1·26)	0·81
	Standard group	134	1626·37 (1·12)	ref	..	194	1489·91 (1·10)	ref	..
Yellow fever PRNT_90_ titres (4 weeks post-vaccination)									
	Intensive group	133	146·34 (1·11)	0·90 (0·67–1·20)	0·46	184	146·46 (1·10)	0·98 (0·76–1·27)	0·89
	Standard group	134	163·11 (1·11)	ref	..	194	149·18 (1·09)	ref	..
*S* Typhi O:LPS-specific IgG (4 weeks post-vaccination), EU/mL									
	Intensive group	132	508·54 (1·09)	1·20 (0·91–1·57)	0·19	182	527·17 (1·08)	1·11 (0·88–1·40)	0·37
	Standard group	132	424·92 (1·11)	ref	..	190	473·96 (1·09)	ref	..
HPV-16-specific IgG (4 weeks post-vaccination), EU/mL									
	Intensive group	133	84·45 (1·11)†	0·71 (0·54–0·94)†	0·017 †	184	94·67 (1·09)	0·82 (0·65–1·03)	0·091
	Standard group	134	119·02 (1·10)†	ref	..	194	115·83 (1·08)	ref	..
HPV-18-specific IgG (4 weeks post-vaccination), EU/mL									
	Intensive group	133	479·43 (1·09)	0·83 (0·66–1·05)	0·11	184	503·38 (1·07)	0·93 (0·76–1·12)	0·44
	Standard group	134	577·93 (1·09)	ref	..	194	543·29 (1·07)	ref	..
Tetanus toxoid-specific IgG (24 weeks post-vaccination), IU/mL									
	Intensive group	123	4·92 (1·09)	1·09 (0·85–1·41)	0·51	168	4·80 (1·08)	1·16 (0·95–1·43)	0·15
	Standard group	100	4·51 (1·10)	ref	..	146	4·12 (1·07)	ref	..
Diphtheria toxoid-specific IgG (24 weeks post-vaccination), IU/mL									
	Intensive group	123	1·60 (1·04)	0·93 (0·82–1·05)	0·23	168	1·66 (1·04)	0·97 (0·87–1·07)	0·51
	Standard group	100	1·73 (1·05)	ref	..	146	1·72 (1·04)	ref	..
**Secondary endpoint analysis**									
BCG-specific IFNγ (52 weeks post-vaccination), SFUs per 1 million PBMCs									
	Intensive group	122	122·04 (1·07)	1·02 (0·83–1·26)	0·83	168	127·89 (1·07)	1·05 (0·88–1·25)	0·61
	Standard group	101	119·23 (1·09)	ref	..	153	122·04 (1·06)	ref	..
Yellow fever PRNT_50_ titres (week 52, 48 weeks post-vaccination)									
	Intensive group	118	2005·37 (1·12)	1·25 (0·91–1·71)	0·17	165	1968·94 (1·10)	1·11 (0·86–1·44)	0·41
	Standard group	107	1607·69 (1·12)	ref	..	157	1768·64 (1·10)	ref	..
Yellow fever PRNT_90_ titres (week 52, 48 weeks post-vaccination)									
	Intensive group	118	226·93 (1·12)	1·25 (0·90–1·75)	0·18	165	214·05 (1·10)	1·12 (0·86–1·48)	0·40
	Standard group	107	181·06 (1·13)	ref	..	157	190·61 (1·10)	ref	..
*S* Typhi O:LPS-specific IgG (week 52, 48 weeks post-vaccination), EU/mL									
	Intensive group	118	174·90 (1·11)	1·10 (0·81–1·50)	0·54	164	192·81 (1·09)	1·11 (0·86–1·43)	0·42
	Standard group	106	158·96 (1·12)	ref	..	154	173·92 (1·10)	ref	..
HPV-16-specific IgG (week 52, 48 weeks post-vaccination), EU/mL									
	Intensive group	110	392·31 (1·13)	0·84 (0·59–1·19)	0·33	151	399·90 (1·11)	0·91 (0·68–1·21)	0·50
	Standard group	96	466·99 (1·14)	ref	..	139	441·72 (1·11)	ref	..
HPV-18-specific IgG (week 52, 48 weeks post-vaccination), EU/mL									
	Intensive group	110	761·32 (1·12)	0·95 (0·71–1·29)	0·75	151	819·11 (1·10)	1·03 (0·80–1·32)	0·82
	Standard group	96	798·40 (1·11)	ref	..	139	796·39 (1·09)	ref	..

CAA=circulating anodic antigen. EU=ELISA unit. HPV=human papillomavirus. IU=international unit. O:LPS=O-lipopolysaccharide. PMBC=peripheral blood mononuclear cells. PRNT_50_=plaque reduction neutralising reference tests, for the reciprocal of the last plasma dilution that reduced by 50%. PRNT_90_=plaque reduction neutralising reference tests, for the reciprocal of the last plasma dilution that reduced by 90%. SFU=spot-forming units. *S* Typhi=*Salmonella enterica* serovar Typhi. Ty21a=live oral typhoid vaccine.

* The different values for n arise because of loss to follow-up and because for each vaccine-specific outcome, we excluded participants who did not receive the vaccine corresponding to that outcome from the analysis. For the *S* Typhi response, we excluded participants who did not receive any of the three Ty21a vaccine doses.

† Statistically significant results.

The effect of intensive versus standard praziquantel administration on vaccine-specific responses in the primary analysis population, stratified by sex, is shown in table 3. Intensive praziquantel administration significantly improved the week 8 *S* Typhi O:LPS-specific response in female participants (GMR 1·57 [1·02–2·41], p=0·039) compared with standard treatment. For HPV, responses to both HPV-16 and HPV-18 were lower in male participants than female participants, and intensive praziquantel administration had a significant adverse effect on the priming HPV-16-specific IgG response in males only (GMR 0·64 [0·47–0·88], p=0·006). However, the p_interaction_ was greater than 0·05 for both analyses (0·46 for HPV-16 and 0·84 for HPV-18).

**Table 3:** Effect of intensive versus standard praziquantel administration on vaccine responses, stratified by sex

	**Female**				**Male**				**p_interaction_**
	n	Geometric mean (SE)	Geometric mean ratio (95% CI)	p value	n	Geometric mean (SE)	Geometric mean ratio (95% CI)	p value	
**BCG-specific IFNγ (8 weeks post-vaccination)**									
Intensive group	43	192·39 (1·14)	1·07 (0·78–1·49)	0·67	67	221·05 (1·09)	1·23 (0·94–1·61)	0·125	0·52
Standard group	45	179·12 (1·11)	ref	..	65	179·63 (1·11)	ref	..	..
**Yellow fever PRNT**_50_**titres (4 weeks post-vaccination)**									
Intensive group	55	1298·51 (1·18)	0·74 (0·45–1·19)	0·21	78	1558·62 (1·15)	1·01 (0·68–1·50)	0·963	0·32
Standard group	52	1765·16 (1·20)	ref	..	82	1544·07 (1·15)	ref	..	..
**Yellow fever PRNT**_90_**titres (4 weeks post-vaccination)**									
Intensive group	55	140·00 (1·18)	0·83 (0·52–1·33)	0·44	78	150·98 (1·14)	0·95 (0·66–1·37)	0·764	0·66
Standard group	52	168·66 (1·18)	ref	..	82	159·68 (1·15)	ref	..	..
***S* Typhi O:LPS-specific IgG (4 weeks post-vaccination)**									
Intensive group	54	555·84 (1·14)*	1·57 (1·02–2·41)*	0·039*	78	478·17 (1·13)	1·00 (0·71–1·43)	0·986	0·11
Standard group	51	354·05 (1·19)*	ref	..	81	476·65 (1·14)	ref	..	..
**HPV-16-specific IgG (4 weeks post-vaccination)**									
Intensive group	55	140·24 (1·18)	0·79 (0·49–1·26)	0·31	78	59·03 (1·13)*	0·64 (0·47–0·88)*	0·006*	0·46
Standard group	52	178·58 (1·19)	ref	..	82	92·03 (1·11)*	ref	..	..
**HPV-18-specific IgG (4 weeks post-vaccination)**									
Intensive group	55	646·73 (1·16)	0·84 (0·56–1·27)	0·41	78	388·20 (1·09)	0·80 (0·62–1·04)	0·101	0·84
Standard group	52	766·97 (1·16)	ref	..	82	482·99 (1·10)	ref	..	..
**Tetanus toxoid-specific IgG (24 weeks post-vaccination)**									
Intensive group	45	4·22 (1·16)	1·05 (0·69–1·60)	0·81	78	5·37 (1·12)	1·12 (0·81–1·55)	0·489	0·82
Standard group	100	4·51 (1·10)	ref	..	146	4·12 (1·07)	ref	..	..
**Diphtheria toxoid-specific IgG (24 weeks post-vaccination)**									
Intensive group	45	1·61 (1·06)	0·85 (0·69–1·05)	0·12	78	1·60 (1·05)	0·97 (0·83–1·13)	0·699	0·31
Standard group	34	1·90 (1·10)	ref	..	66	1·65 (1·06)	ref	..	..

HPV=human papillomavirus. O:LPS=O-lipopolysaccharide. PRNT_50_=plaque reduction neutralising reference tests, for the reciprocal of the last plasma dilution that reduced by 50%. PRNT_90_=plaque reduction neutralising reference tests, for the reciprocal of the last plasma dilution that reduced by 90%. *S* Typhi-*Salmonella enterica* serovar Typhi.

* Statistically significant results.

The effect of intensive versus standard praziquantel administration on vaccine-specific responses in participants who had *S mansoni* CAA of 30 pg/mL or higher at baseline but had reduced *S mansoni* CAA concentrations to under 30 pg/mL by the time of first vaccination (week 0 for BCG, YF-17D, Ty21a, and HPV; week 28 for tetanus–diphtheria) in the intensive treatment group is shown in appendix 2 (p 5). Analysis showed a likely benefit of intensive praziquantel administration on the week 8 BCG-specific IFNγ ELISpot response (GMR 1·29 [95% CI 0·98–1·71], p=0·07), and an adverse effect on the HPV-16 (0·60 [0·41–0·87], p=0·007) and HPV-18 (0·75 [0·56–1·00], p=0·053) responses compared with standard treatment.

AUC calculations did not show any significant differences between trial groups for vaccine responses at either the primary endpoint at week 8 or the secondary endpoint at week 52 (appendix 2 pp 5–6).

Proportions of participants who were seropositive (PRNT_50_ titre ≥10) following YF-17D vaccination, had protective tetanus toxoid-specific IgG levels (≥0·1 IU per mL) post tetanus–diphtheria vaccination, or had 4-fold or greater increase in *S* Typhi O:LPS-specific IgG following Ty21a vaccination, were comparable between intensive and standard intervention groups (appendix 2 p 6). Furthermore, considering HPV-16-specific and HPV-18-specific responses after priming and boosting doses, there was no difference in the effect of intensive intervention on the priming versus the boosting response (appendix 2 pp 6–7).

## Discussion

We found that treating schistosomiasis with an intensive praziquantel administration regimen resulted in a sharp decline in infection intensity, assessed by CAA concentration, and a substantial—but more modest—change in *S mansoni* infection prevalence. Despite the effect on the parasite, intensive praziquantel administration showed no statistically significant improvement in vaccine responses at the primary endpoints (8 weeks post-BCG vaccination; 4 weeks post-yellow fever and oral typhoid vaccination; and 24 weeks post tetanus and diphtheria vaccination) when analysis was confined to the group shown to be infected at baseline. Intensive praziquantel administration also resulted in a lower HPV-16-specific antibody response to the first vaccine dose. However, when the whole trial population was considered, intensive praziquantel administration resulted in an improved response to BCG vaccination at week 8.

Our trial took place in the context of the overall POPVAC programme of interrelated trials, which showed substantial differences in responses to most vaccines between the three settings: urban, rural malaria-endemic, and rural schistosomiasis-endemic.^28^ Compared to the urban setting, children in the rural, schistosomiasis-endemic setting showed lower responses to YF-17D, Ty21a, and tetanus vaccines, and a different dynamic for BCG with higher baseline ELISpot responses, but a similar peak response at week 8 and waning response at week 52 following vaccination. This aligns with the findings of our systematic review, where both animal models and observational studies in humans indicate an association between schistosome infection and impaired responses to several vaccines.^12^ Despite this cumulative evidence for an adverse effect of schistosome exposure, the POPVAC A trial results further accord with earlier trials in showing a minimal effect, if any, of treating schistosomiasis on most vaccine responses.

However, there was evidence suggesting a beneficial effect of intensive praziquantel administration on the BCG response 8 weeks post-vaccination. This was seen only in the whole trial population; it is quite likely that children with a CAA result below 30 pg/mL were actually infected, as this is not the most sensitive version of the assay,^25^ and therefore able to benefit from treatment. Our ELISpot assays measure circulating effector cells,^29^ and it would be appropriate to investigate whether there is any durable effect on memory responses, or whether there is an effect on cytokine levels from these cells, using flow cytometry, or on functional parameters such as mycobacterial growth inhibition. This possible benefit for BCG could be a chance finding, but it accords with animal models and our meta-analysis which suggests particular sensitivity of BCG to the immunomodulating effects of helminths.^12^ Additionally, BCG was the only vaccine for which we assessed a cellular response, and it would be interesting to explore whether cellular, compared to humoral, immune responses are more susceptible to the immunomodulating effects of helminths, and responsive to their treatment.

By contrast, there was evidence of a modest adverse effect of intensive praziquantel administration on the HPV-16 response. In the primary analysis, only the peak priming response for HPV-16 was affected, with no effect after boosting, nor was there an effect on the HPV-18 response. In the sex-stratified analysis it was noted that responses to both HPV-16 and HPV-18 were lower in male participants than female participants, consistent with a recent meta-analysis,^30^ and there was some evidence that the adverse effect of intensive praziquantel administration on HPV-16 might be more prominent in male participants (perhaps related to the weaker overall response). Taken together, these findings suggest that careful monitoring of HPV-specific responses and efficacy should be undertaken as the HPV vaccination schedule transitions to single dose and is rolled out to male children.

Our trial design and sample size were strong, providing results on a broad range of vaccines applicable to schoolchildren in schistosomiasis-endemic settings, a key target group for vaccinations, including for the prevention of tuberculosis in adolescents and young adults, and sexually transmitted infections and diseases. Our study has some limitations. The lower-than-expected rate of CAA positivity reduced power for our primary analysis. We used a height pole to estimate the praziquantel dose; this might result in sub-optimal doses for individuals with a high BMI. However, the prevalence of overweight in our study was less than 2%, and none of the study participants were obese. The COVID-19 pandemic-associated lockdown and school closures in Uganda meant that we missed one planned praziquantel and albendazole treatment at week 32, which was also the original primary endpoint for tetanus–diphtheria, 4 weeks after the administration of these vaccines. Furthermore, although intensive praziquantel administration reduced *S mansoni* infection, it did not eliminate the parasite, allowing the possibility of continuing effects of low-level active infection. Thus, comparisons between trial groups do not correspond precisely to an assessment of full *S mansoni* removal. However, data from participants in the intensive treatment group who had *S mansoni* CAA of 30 pg/mL or higher at baseline, but had reduced *S mansoni* CAA concentrations to under 30 pg/mL by the time of first vaccination versus participants who were still infected in the standard group (appendix 2 p 5) supports our main observations. In this group there was still a probable benefit of intensive praziquantel administration on the week 8 BCG response, an adverse effect on the HPV responses, but no effect on other vaccine responses. It is worth noting the possibility of undetected *S mansoni* infection at week 8, masking the true effect of intensive praziquantel administration in this group of participants, as we had results from the CAA assay but not PCR at that timepoint. A substantial proportion of our study population were also infected with other parasitic infections. However, we do not expect any immunomodulatory effects of these parasites to bias the effect of praziquantel administration on vaccine responses as this was a randomised trial; prevalence of the parasitic infections that we assessed was balanced between the two trial groups.

In conclusion, animal studies and observational studies indicate that impaired responses to several vaccines are associated with *S mansoni*, and our POPVAC urban–rural comparisons^28^ showed that some vaccine responses are reduced in *S mansoni*-endemic settings. Here, we show evidence suggesting that praziquantel administration improves the BCG-specific response, but not responses to other vaccines. Our suggestive results regarding an improvement in BCG response following praziquantel administration merit further exploration of memory responses, functional assays, and underlying mechanisms, in the light of literature supporting this effect. As our trial focused on current *S mansoni* infection and praziquantel intervention, future emphasis should be on understanding whether there are longer-term effects of chronic or repeated helminth infections on vaccine response.

### POPVAC trial team

### Contributors

### Equitable partnership declaration

### Data sharing

The de-identified individual participant data that underlie the results reported in this article are stored in a non-publicly available repository (London School of Hygiene & Tropical Medicine [LSHTM] Data Compass), together with a data dictionary. Data are available on request. Researchers who would like to access the data may submit a request through LSHTM Data Compass, detailing the data requested, the intended use for the data, and evidence of relevant experience and other information to support the request. The request will be reviewed by the Principal Investigator in consultation with the Medical Research Council/Uganda Virus Research Institute (MRC/UVRI) and LSHTM data management committee, with oversight from the UVRI and LSHTM ethics committees. In line with the MRC policy on data sharing, there will have to be a good reason for turning down a request. Patient Information Sheets and consent forms specifically referenced making anonymised data available and this has been approved by the relevant ethics committees. Researchers given access to the data will sign data sharing agreements which will restrict the use to answering pre-specified research questions.

## Declaration of interests

GN and AME report grants from the Wellcome Trust. GN reports funding from the EDCTP2 programme supported by the EU. AME and SC report funding from UK Medical Research Council (MRC) for conduct of the study. AME reports funding from the US National Institutes for Health, Science for Africa Foundation, the Royal Society, and DELTAS Africa, outside the submitted work. AME and AN report support from UK National Institute of Health and care Research (NIHR). AME further reports support from the Serum Institute of India, Uganda National Expanded Program on Immunization, and Emergent BioSolutions for conduct of the study. All other authors declare no competing interests.

## Supplementary Materials

Luganda translation of the abstract

Supplementary appendix 2

Equitable Partnership Declaration
