# Supplementary material for: The effect of intensive praziquantel administration on vaccine-specific responses among schoolchildren in Ugandan schistosomiasis-endemic islands (POPVAC A): an open-label, randomised controlled trial
Source: Lancet Glob Health. 2024 Oct 16;12(11):e1826–37. doi: 10.1016/S2214-109X(24)00280-8 (PMC11483245; doi:10.1016/S2214-109X(24)00280-8)
Supplement: Luganda translation of the abstract [file mmc1.pdf]

THE LANCET
Global Health

Supplementary appendix 1

This translation in Luganda was submitted by the authors and we reproduce it as supplied. It has not been peer reviewed. The Lancet's editorial processes have only been applied to the original in English, which should serve as reference for this manuscript.

Ekiwandiiko kino ekikyuse mu lulimi Oluganda kyaweereddwayo abawandiisi b'ennyini era tukifulumizza nga bwe baakituwadde. Tekinnakubaganyizibwako birowoozo abakugu abalala. Okusunsula kwa Lancet kukoleddwa ku ekyo eky'Olungereza kyokka nga bwe kyafulumizibwa era kye kirina okujulizibwako ku kiwandiiko kino.

Supplement to: Nkurunungi G, Nassuuna J, Natukunda A, et al. The effect of intensive praziquantel administration on vaccine-specific responses among schoolchildren in Ugandan schistosomiasis-endemic islands (POPVAC A): an open-label, randomised controlled trial. *Lancet Glob Health* 2024; **12:** e1826–37.

Mu bufunze

Ennyanjula

Engeri omubiri gye gwanukulamu ku ddagala erigema endwadde ekyukakyuka okusinziira ku bantu ab'enjawulo era ebiseera ebisinga amaanyi gaalyo gakendeera mu byalo, mu ba mufuna mpola. Ensonga lwaki kiri bwekiti tezinnategeerekeka bulungi. Enkyukakyuka mu kulwanyisa endwadde ezireetebbwa ebiwuka ziyinza okuba nga zirina engeri gye zivunaanyizibwamu. Twakola okuteebereza nti obuwuka obuleeta *Bilihaziya* (*Schistosoma mansoni*) bunyigiriza enkola y'eddagala erigema eritalina kakwate ku linnaalyo era okulemesa okwo kusobola okukyusibwa, waakiri ekitundu, nga tuyita mu kujjanjaba ennyo Bilihaziya.

Enkola

Twakola okugezesa okwokweyambisa eddagala nga tekuliimu bya kukwekereza, mu kunoonyereza okwokuwa obujjanjabi emirundi emingi n'okwabulijjo okumanyiddwa okw'okulwanyisa *Schistosoma mansoni* mu baana abali mu myaka egisoma (emyaka 9-17) ku bizinga by'e Koome mu Uganda. Abaana mu ngeri etali ya kyekubiira baateekebwa mu kibinja omwaali okuweebwa obujjanjabi emirundi emingi n'ekibinja omwali okujjanjabwa okwa bulijjo okumanyiddwa okulwanyisa Sm nga tukozesa engeri y'okweroboza okuteeka abeetabi mu bibinja eya kimu ku kinaakyo eyava mu kyuma ki kalimagezi . Abeetabi abali ku ludda lw'okujjanjaba emirundi emingi baafuna doozi ssatu (40mg/kg) ez'eddagala erirwanyisa Bilihaziya mu wiiki bbiri ezeesudde nga tebannagemebwa mulundi gusooka mu wiiki 0, n'oluvannyuma ne batandika okuweebwa buli luvannyuma lwa myezi esatu. Abeetabi ku luuyi olwa bulijjo, bajjanjabibwa oluvannyuma lwa wiiki ey'o 8. Abeetabi baaweebwa eddagala BCG erigema akafuba (Serum Institute of India, Pune,India) ku wiiki 0; nebagemwa omusujja gw'enkaka (Sanofi Pasteur, Lyon, France), omusujja gwo mu byenda (PaxVax, London, UK), Kookolo w'omumwa gwa nabaana HPV (Merck, Rahway, NJ, USA) ku wiiki 4; eryongera HPV, tetanus/diphtheria (Serum Institute of India) ku wiiki 28. Ebyavaamu ebikulu kwali kulaba engeri emibiri gyegyanukulamu ku ddagala erigema mu wiiki ey'omunaana okuggyako erya tetanus/diphtheria ery'ekenneenyezebwa ku wiiki eya 52. Okwekenneenya okusookerwako kwaali mu bantu abaali beetabye mu kugezesebwa abaali bakwatibwa *Schistosoma mansoni* ku ntandikwa. Okuba n'obuwuka bwa

Schistosoma mansoni kwakolebwa mu ngeri ya kaddanyuma nga tukebera obwenkulumu bw'akawuka kano mu musaayi oba circulating anodic antigen (CAA) n'ery'omu bubi nga tukozesa PCR. Omusomo guno gwawandiikibwa ku ISRCTN Registry (ISRCTN60517191) era gwaggwa.

Ebyazuulwa

Wakati w'ennaku z'omwezi 9 Ogw'omusanvu ne 14 Ogw'omunaana 2019, twayingiza abeetabi 478 nga be baana 239 mu buli kibinja, Abeetabi 276 (58%) baali balenzi ate nga 202 (42%) baali bawala. Mu baalina obulwadde bwa Bilihaziya mu ntandikwa (171(72%) baagwa mu kibinja eky'okufuna eddagala emirundi emingi ate nga 164 (69%) baali ku b'eddagala erya bulijjo) okuweebwa kw'eddagala lya Bilihaziya emirundi emingi kwagenda kweyongera okukendeza obunyiinyiitivu bw'obulwadde buno ng'okugemwa tekunnabaawo (median [IQR] CAA, pg/ml, 30[7;223] vs 1317[243;8562], $p<0.001$) nga tugeraageranya n'abaafuna eddagala mu ngeri ya bulijjo. Okuweebwa kw'eddagala lya Bilihaziya emirundi emingi kwakendeeza engeri omubiri gyegwanukulamu ku kugema ne HPV-16 ku wiiki ey'o 8 (omuwendo omukkati 0.71 (95%CI 0.54-0.94), $p=0.017$), naye tekyaalaga njawulo ku byavaamu ebirala. Mu beetabi bonna (nga tofuddeeyo ku bwe bayimiridde ku bya *Schistosoma mansoni* mu ntandikwa), okujjanjaba n'eddagala lya Bilihaziya emirundi emingi kwayongera nnyo okukola obulungi n'addala ku baagemwa ne BCG oluvanyuma lwa wiiki ey'o 8, mu kwanukula kw'omubiri IFNɣ: (1.20 (1.01-1.43), $p=0.038$). Obuzibu obumanyiddwa obuva ku kumira praziquantel bwaloopebwa nnyo mu kibinja eky'okukozesa eddagala emirundi emingi. Tewaaliwo buzibu bwa maanyi obwawandiikibwa mu kibinja kyonna.

Entaputa

Tulaga obujulizi nti okuwa eddagala lya Bilihaziya kwongeza mu ngeri obutoffaali gyebwanukulamu ku ddagala erigema erya BCG naye tekwongera ku kwanukula kw'omubiri nga kiva ku ddagala eddala erigema. Wadde nga waliwo obujulizi obw'okwetegereza nti ebiwuka bikosa okwanukula kw'okugema, ebivuddemu bino biraga emigaso mitono egy'amangu egy'okukendeeza ku bungi bwebiwuka. Ebiva mu kukendeeza ebiwuka mu bbanga eggwanvu bisaana okunoonyezebwa

Abataddemu ensimbi

UK Medical Research Council (MR/R02118X/1)
